# Evidence for Direct Involvement of the Capsid Protein in HIV Infection of Nondividing Cells

**DOI:** 10.1371/journal.ppat.0030156

**Published:** 2007-10-26

**Authors:** Masahiro Yamashita, Omar Perez, Thomas J Hope, Michael Emerman

**Affiliations:** 1 Division of Human Biology, Fred Hutchinson Cancer Research Center, Seattle, Washington, United States of America; 2 Department of Microbiology and Immunology, University of Illinois at Chicago, Chicago, Illinois, United States of America; 3 Department of Cell and Molecular Biology, Feinberg School of Medicine, Northwestern University, Chicago, Illinois, United States of America; Aaron Diamond AIDS Research Center, United States of America

## Abstract

HIV and other lentiviruses can productively infect nondividing cells, whereas most other retroviruses, such as murine leukemia virus, require cell division for efficient infection. However, the determinants for this phenotype have been controversial. Here, we show that HIV-1 capsid (CA) is involved in facilitating HIV infection of nondividing cells because amino acid changes on CA severely disrupt the cell-cycle independence of HIV. One mutant in the N-terminal domain of CA in particular has lost the cell-cycle independence in all cells tested, including primary macrophages. The defect in this mutant appears to be at a stage past nuclear entry. We also find that the loss of cell-cycle independence can be cell-type specific, which suggests that a cellular factor affects the ability of HIV to infect nondividing cells. Our data suggest that CA is directly involved at some step in the viral life cycle that is important for infection of nondividing cells.

## Author Summary

HIV and related viruses are unusual among retroviruses in their ability to replicate independently of cell-cycle progression of target cells. However, the determinants of this phenotype have been controversial. Here, we identified mutations on the surface of the capsid (CA) protein that reduce the ability of HIV to infect nondividing cells. These mutations also confer cell-cycle dependency on HIV, even in dividing cells. Interestingly, some CA mutants lose cell-cycle independence only in certain cell types. Thus, these findings suggest that a cellular factor targeting CA regulates HIV-1 infection in nondividing cells. Surprisingly, these mutations do not appear to affect nuclear localization of viral genomes, which points to a novel regulation of the cell-cycle independence of HIV by the CA protein.

## Introduction

One of the properties that set HIV-1 and other lentiviruses apart from most of the other retroviruses is the ability to infect cells independent of the cell cycle [1,2]. This ability allows HIV-1 to propagate in nondividing cells in vivo such as resting CD4+ T cells [3] and terminally differentiated macrophages [4]. On the other hand, other retroviruses, such as murine leukemia virus (MLV), require cell-cycle progression to achieve productive infection [5,6].

There has been considerable controversy over the determinants of HIV infectivity in nondividing cells, with most studies concentrating on presumed determinants for nuclear import [2,7]. However, we recently showed that none of the previously identified karyophilic elements in the HIV genome are necessary for HIV to infect nondividing cells [8]. Rather, we demonstrated that the retroviral capsid (CA) protein is a major determinant for retrovirus infection in nondividing cells because an HIV-based chimeric virus with MLV CA does not infect nondividing cells [8]. Nonetheless, it was not clear whether or not HIV CA was required to infect nondividing cells, or whether we had transferred a negative regulator of nuclear entry from MLV onto HIV. The present study was designed to determine whether HIV CA plays a direct role in the ability of this virus to infect nondividing cells.

The CA protein is a major structural protein that constitutes viral cores, and also plays a role in the early stages of infection (reviewed in [9]). Soon after virus entry into the target cell, incoming virions disassemble their cores in the cytoplasm (uncoating). However, it is not well understood exactly how the uncoating process takes place in acutely infected cells and which cellular factors may be involved [10,11]. Moreover, the uncoating steps may be different between HIV and MLV since most of the CA proteins of HIV dissociate from nucleoprotein complexes of incoming virions [10–17], whereas a large amount of CA remains bound to intracellular complexes of MLV after infection [18–20]. Therefore, one plausible hypothesis is that the difference in the uncoating process may influence the fate of retrovirus infection in nondividing cells by affecting further downstream events (nuclear import and integration) [21].

Here, we show that mutations in HIV CA can specifically reduce the infectivity of HIV in nondividing cells, and recapitulate the need for cell-cycle progression as seen for MLV. Furthermore, cell-cycle independence of most of the mutants is lost only in a particular cell type, which suggests that a cellular factor limits their replication in nondividing cells. We show that reverse transcription and nuclear import of these mutants proceed normally in nondividing cells. Finally, we show that, contrary to expectations, the kinetics of uncoating of the bulk of CA from the incoming virus cores does not correlate with the ability to infect nondividing cells. However, a functional assay for CA association with the reverse transcriptase complex (RTC) suggests that prolonged association of some CA with the RTC is associated with a loss of cell-cycle independence. These results suggest a direct role for CA that is important for the ability of HIV to infect nondividing cells.

## Results

### Mutations in CA That Decrease the Ability of HIV-1 to Infect Nondividing Cells

**Figure 1:**
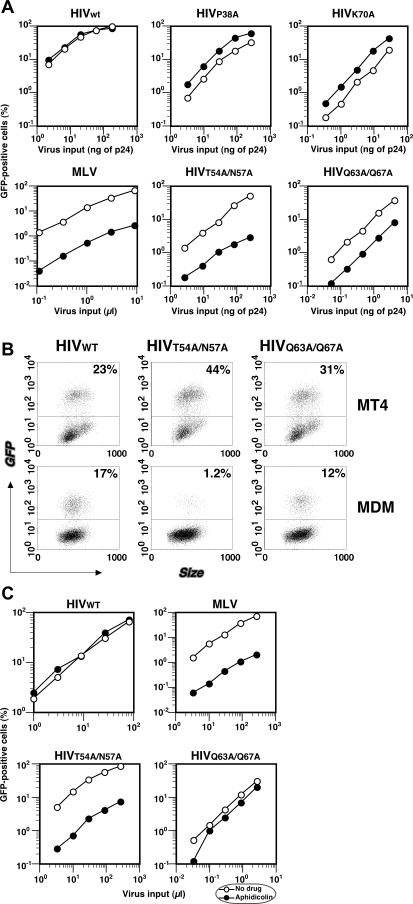
HIV CA Mutants Require Cell Division for Efficient Infection (A) Dose-independent restriction of several CA mutants in nondividing cells. Nondividing cells were prepared by treatment of HeLa cells with aphidicolin and then infected with increasing amount of GFP-encoding viruses. Virus infectivity was measured by quantifying GFP-positive cells 2 d after infection. WT HIV-1 and MLV were used as controls. Open circles indicate infections with cycling cells and closed circles indicate infections with nondividing cells. (B) Infection of MDMs with HIV-1. The WT strains together with CA mutants T54A/N57A and Q63A/67A were used to infect a human T cell line (MT4: upper panels) or MDMs (lower panels). Ten times more virus was used in MDMs, which are not as permissive as MT4 cells to HIV-1 infection. Expression of GFP, which is encoded by the reporter virus constructs, was examined 2 or 3 d after infection. The % of GFP-positive cells is indicated in the upper half of each flow cytometry panel. The data are representative of three independent experiments using at least three different donor PBMCs. (C) Viral infectivity of CA mutants in HOS cells. Cell-cycle requirement for virus infection was examined by infectivity in dividing and nondividing HOS cells with WT or CA mutants encoding the GFP gene. Nondividing cells were prepared by treatment of aphidicolin (2 μg per ml) and the chemical was added throughout the experiment. Open circles indicate infections with cycling cells and closed circles indicate infections with nondividing cells. Shown here are the data that represent at least two independent experiments.

A panel of HIV CA mutations was created and tested for their ability to infect nondividing cells (Table 1). These mutations were introduced in amino acids exposed on the surface of CA that had been previously characterized as mutations that did not have severe effects on virus assembly or budding, but decreased viral infectivity [22]. We introduced each mutation onto an HIV-1 provirus with a deletion in envelope (i.e., a single-round vector) that encodes either luciferase or green fluorescent protein (GFP) in place of the nef gene [21]. In our initial screen for infection of nondividing cells, we arrested HeLa cells in S-phase by addition of aphidicolin to the culture. As expected, the titer of MLV, which requires cell-cycle progression for productive infection, was reduced by 50-fold in nondividing cells relative to dividing cells, while infectivity of wild-type (WT) HIV-1 is equivalent in both dividing and nondividing cells (Figure 1A; Table 1). In contrast, CA mutants (T54A/N57A and Q63A/Q67A) were reduced in nondividing cells compared to dividing cells by 5- to 10-fold (Figure 1A; Table 1). The reduced infectivity of these CA mutants in nondividing cells is independent of multiplicity of infection, as the reduction in infectivity can be observed over a 3-log range of viral input (Figure 1A).

**Table 1:**
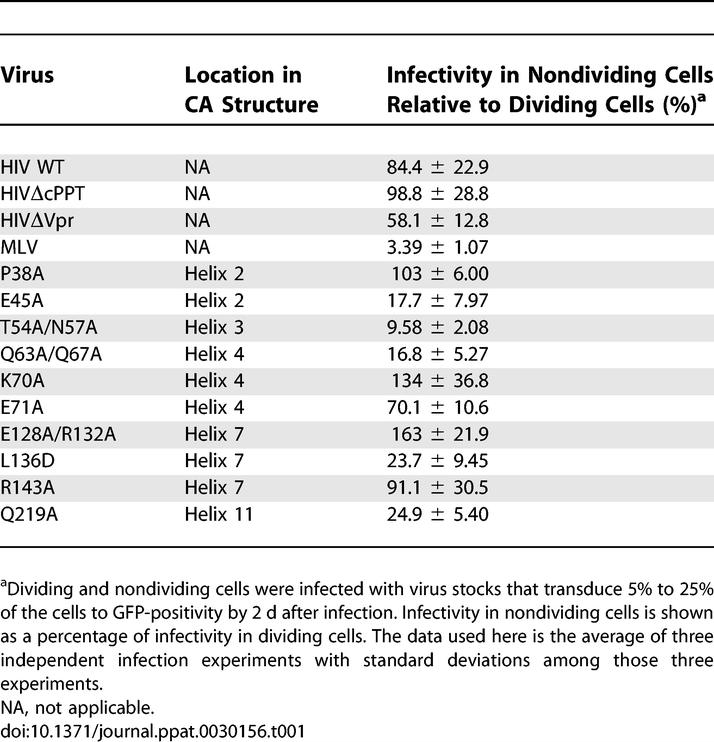
HIV Mutants Tested for Their Ability to Infect Nondividing Cells

The decreased infectivity of the CA mutants in nondividing cells is more severe than previously characterized mutations in the *vpr* gene and the central polypurine tract [8], which had little effect on the ability of HIV to infect nondividing cells (Table 1). Importantly, the reduction of virus infectivity by itself does not necessarily cause the defect in the cell-cycle independence for infection, because some CA mutants that exhibit reduced overall infectivity still maintain the same property to infect nondividing cells as efficiently as dividing cells (for example, the P38A and K70A mutations; Figure 1A). In total, five separate mutants (E45A, T54A/N57A, Q63A/Q67A, L136D, and Q219A) were identified that exhibit at least a 4-fold decrease in the ability to infect nondividing cells (Table 1).

We also infected monocyte-derived macrophages (MDMs), a nondividing cell type that is a natural target of HIV infection. Viruses were first normalized for infectivity by titrating them on MT4 cells and equivalent MT4 cell infectious doses were used to infect macrophages. Figure 1B shows representative flow cytometry data showing that one of the mutants, T54A/N57A, is indeed very defective in nondividing macrophages. Namely, while infectivity of WT HIV-1 in MDMs (17%) was basically similar to that in MT4 cells (23%), the infectivity of T54A/N57A in MDMs (1.2%) was much lower than in MT4 cells (44%), as shown in Figure 1B.

Interestingly, the Q63A/Q67A mutant is affected only slightly in nondividing MDMs (Figure 1B). Thus, we also tested the ability of CA mutants to infect another cell line, HOS cells, that were dividing or nondividing. We found that the T54A/N57A mutant could not infect nondividing HOS cells, although it could infect dividing HOS cells (Figure 1C). However, the Q63A/Q67A mutant (Figure 1C) and all of the other CA mutants other than T54A/N57A (not shown) could infect nondividing HOS cells just as well as it could infect dividing HOS cells. These results indicate that there is cell-type specificity to the ability of most of the mutants to infect nondividing cells. Therefore, these findings suggest the presence of cellular factors that differentially regulate virus infectivity in non-cycling cells, but point to T54A/N57A as a region of CA that is important for infection of nondividing cells in diverse cell types.

### Passage through Mitosis Alleviates Replication Block of HIV CA Mutants

**Figure 2:**
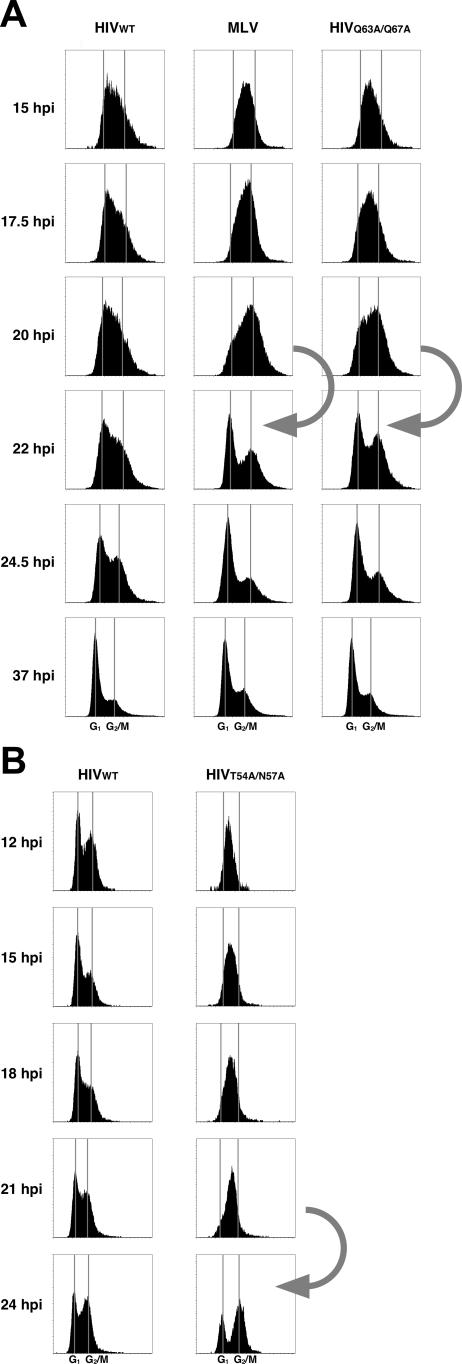
HIV-1 CA Mutants Resemble MLV in Their Cell-Cycle Requirements for Transduction HeLa cells were infected with GFP-encoding viruses HIV-1, MLV, and HIV-1 CA mutants (A) Q63A/Q67A or (B) T54A/N57A. The cell-cycle profiles of the GFP-positive populations of cells are shown. The *y*-axis in each plot is the number of cells and the *x*-axis is the cell-cycle position and the approximate positions of cells in G1 (left vertical line) and G2/M (right vertical line) are indicated. HIV-1 in (A) carries the mutated *vpr* gene, whereas the virus in (B) contains the intact *vpr* gene. The arrow indicates the progression of the transduced populations infected with MLV or with the HIV-1 CA mutants from G2/M to G1. Note that WT HIV remains asynchronous in the cell cycle throughout the experiment.

MLV can only infect cells that pass through mitosis [5,6]. We asked if the HIV CA mutants that failed to infect nondividing cells had a requirement for mitosis similar to MLV. To determine if there is a cell-cycle stage necessary for productive transduction, we adapted a previously described method that does not involve an artificial block of the cell cycle [23]. Actively cycling cells were infected with retroviruses carrying the GFP gene, harvested at different time points after infection, and stained for DNA content with propidium iodide. Cell-cycle analysis of GFP-positive cells (i.e., cells that were successfully transduced with each GFP reporter virus) early after infection revealed that the cell-cycle status of GFP-positive cells in the MLV-infected cell population changes dramatically at different time points after infection, while the cell-cycle status of GFP-positive cells in the HIV-infected cell population remains relatively stable over time (Figure 2). This is likely due to the fact that MLV-transduced cells appear to be synchronized in the cell cycle, because the only transduced cells will be those that have passed through mitosis within a narrow time window after infection. On the other hand, HIV can transduce cells at any stage of the cell cycle, so HIV-transduced cells always appear asynchronous in the cell cycle. Specifically, most of the GFP-positive cells in the MLV-infected cell population were in S-phase 15 h after infection. These GFP-positive cells in MLV-infected cells were in late S-phase 17.5 h after infection, by 20 h they were in G2/M phase, and they completed mitosis from 20 to 22 h post infection (indicated by arrow, Figure 2A, middle). In contrast, GFP-positive cells in the HIV-infected cell population remain asynchronous in their cell-cycle profile at all time points post infection (Figure 2A, left).

We tested two CA mutants, T54A/N57A and Q63A/Q67A, in this assay, and found that cells infected with the HIV-1 CA mutant Q63A/Q67A are nearly indistinguishable from MLV in that they also are in S-phase at 15 h after infection and pass from G2/M to G1 between 20 and 22 h after infection (Figure 2A, right; the transition from G2/M to G1 is indicated by an arrow). We also obtained similar data using another CA mutant (T54A/N57A, Figure 2B). Therefore, these two CA mutants must have cell-cycle requirements for transduction similar to MLV and different from WT HIV. These results suggest that HIV CA mutants that fail to infect nondividing cells, can, like MLV, productively infect cycling cells only after they have passed through mitosis.

### Replication of CA Mutants Was Blocked after Reverse Transcription in Interphase Cells

**Figure 3:**
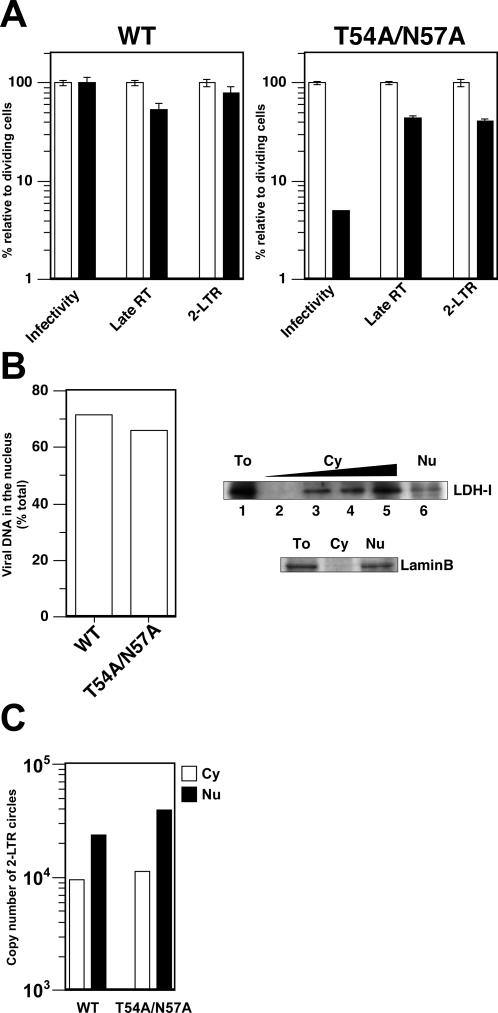
Reverse Transcription and Nuclear Import of Viral DNA in Nondividing Cells (A) Viral DNA synthesis and formation of 2-LTR circles. Dividing (shown in white bars) and nondividing HeLa cells (shown in black bars) prepared by aphidicolin treatment were infected with reporter virus constructs bearing the WT CA or T54A/N57A mutation. Virus infectivity was assessed by quantifying GFP-positive cells 2 d after infection. Relative infectivity is shown as percentage of infectivity in dividing cells. Total DNA was extracted 1 d after infection and used in real-time PCR to measure the copy number of late products of reverse transcription (RT) as well as that of 2-LTR circles. The data is shown as relative values where the amount in dividing cells for each different virus is set at 100%. Infections were done in triplicate and error bars indicate standard deviation. Two independent experiments were performed with similar results. One representative experiment is shown here. (B) Nuclear localization of viral DNA. Nondividing cells infected with either WT HIV-1 or the CA mutant T54A/N57A were separated into cytoplasmic and nuclear fractions (left panel). DNA extracted from each fraction was used as template for real-time PCR to measure newly synthesized viral DNA. The efficiency of nuclear migration of viral DNA was examined by dividing the copy number of viral DNA in nuclear fractions by that in both fractions (cytoplasmic plus nuclear fractions). The data shown here is a representative of two independent experiments; although the ratio of viral DNA associated with nuclear fractions differed between two experiments, the amount of viral DNA associated with nuclear fraction remains the same between WT and the CA mutant. Controls with reverse transcriptase inhibitors showed that contamination by plasmid DNA accounted for less than 1% of the values (not shown). Western blotting analysis was performed to ensure the integrity of subcellular fractionation (right panels). Contamination was checked by checking a cytoplasmic protein, LDH-I (upper lanes) and a nuclar protein, lamin B (lower lanes). To, total cell lysates; Cy, cytoplasmic extract; Nu, nuclear lysates. Twenty migrograms of proteins were loaded on the gel except for the following dilutions. Cytoplasmic extract was diluted by 5-fold; 5-, 25-, and 125-fold dilutions correspond to lanes 4, 3, and 2 (upper lanes). (C) Subcellular localization of 2-LTR circles. Copy numbers of 2-LTR circles in cytoplasmic (white boxes) and nuclear (black boxes) fractions were measured by real-time PCR. The same fractions as in the (B) were used in this PCR assay.

We concentrated our mechanistic studies on the T54/N57A mutant because it had the strongest defect in nondividing cells (Table 1), and because it was affected in all cell types tested (Figure 1). There are ample precedents for mutations in CA that affect reverse transcription [24–30]. Thus, we were interested in knowing whether this mutant had a defect before or after reverse transcription. We therefore used a real-time PCR assay to compare late products of reverse transcription [31] for this mutant compared to those of WT HIV in dividing and nondividing cells. Remarkably, even though the amount of transduction (measured by GFP expression) is reduced to 5% to 10% of the levels of dividing cells (Figure 3A), we found that the T54A/N57A mutant does not have a defect in synthesizing reverse transcription products in nondividing cells. Although we saw a slight decrease in the amount of the late reverse transcription products by T54A/N57A in nondividing cells, the same degree of reduction was also observed for WT HIV. Thus, we conclude that the replication defect of this CA mutant in nondividing cells occurs after reverse transcription.

Another hypothesis to explain why this CA mutant fails to infect nondividing cells is that viral DNA of these mutants is blocked from entering the nucleus before mitosis. Therefore, we investigated nuclear entry by measuring the copy number of 2-LTR circles, a marker of nuclear entry of viral genomes (Figure 3A), and by cell fractionation (Figure 3B). We found very little change in the formation of 2-LTR circles by T54A/N57A in nondividing cells. Although we saw a slight drop of 2-LTR in nondividing cells compared with dividing cells (2.5-fold), this amount of decrease is not likely to explain the reduced infectivity in nondividing cells (19-fold decrease from the infectivity in dividing cells). We obtained substantially similar data for another CA mutant (E45A).

Nuclear entry of viral DNA was also examined by subcellular fractionation followed by real-time PCR for quantification of reverse transcription products. Our cell fractionation protocol was verified by showing that cytoplasmic protein, LDH-1, contaminated the nuclear fraction to less than 4% (Figure 3B, top right), and a nuclear protein, lamin B, could not be detected in the cytoplasmic fraction (Figure 3B, bottom right). Using this assay we found that 70% of the viral DNA of the CA mutant T54A/N57A was associated with the nuclear fraction (Figure 3B). More importantly, the amount of viral DNA associated with the nuclear fraction is similar between WT and T54A/N57A (Figure 3B). Finally, we looked at the localization of the 2-LTR circles in these fractions, and found that in both WT and the T54/N57A mutant, the 2-LTR circles were more abundant in the nuclear fraction than in the cytoplasmic fractions in aphidicolin-treated cells (Figure 3C). These results suggest this CA mutant has the ability to access the nucleus in nondividing cells, and the block to this mutant in nondividing cells may be at a stage post–nuclear entry.

### Uncoating of CA Mutants

**Figure 4:**
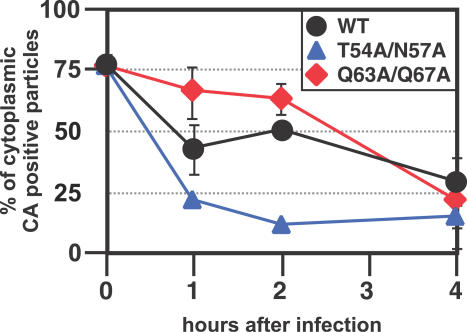
In Situ Analysis of Kinetics of p24 Loss from Cytoplasmic HIV HeLa cells were spinoculated with VSV-g pseudotyped, S15-mCherry, GFP-Vpr labeled HIV-1ΔEnv virions for 2 h at 17 °C. Infection was synchronized by washing off innocula and replaced with 37 °C media. HeLa cells were then fixed and immunostained for p24^CA^ (Cy-5) at the indicated time post infection and imaged. GFP (+) pucnta were then quantified and individually examined for the presence of mCherry and Cy-5 (p24^CA^) signal. The identity of the samples was blinded before the experiment. The percentage of the total number of virions that have stained for p24^CA^ over time following fusion is shown. The 0-h time point represents total number of GFP (+) virions that stained positive for p24^CA^. HIV_WT_ is represented by black lines/circles, the Q63/67A CA mutant by red line/diamonds, and the T54A/N57A CA mutant by blue line/triangles. The results shown are from three independently performed experiments and the standard deviation at each time point is shown.

We previously hypothesized that uncoating of the viral core might be the rate-limiting step for the ability of a retrovirus to infect non-cycling cells [2]. Thus, we sought to examine the uncoating of these CA mutants in vivo. We used a recently developed assay [32] for in situ determination of the CA association with intracellular viral complexes. Briefly, virus is labeled with GFP-Vpr [33] and the membrane is labeled by incorporation of a fluorescent fusion protein that contains the N-terminal 15–amino acid sequence of c-Src (called S15-Cherry) [32]. At various times after infection of HeLa cells, the cells were fixed and stained with an antibody to p24CA. The total complexes that entered the cytoplasm (green spots that lost the membrane dye) were counted, and the number of complexes that contained p24 (not uncoated) was compared to the number of complexes that lost p24 staining (uncoated). The numbers are represented as the % of p24CA positive cytoplasmic particles (Figure 4).

WT virus, as expected, began to lose CA soon after infection (Figure 4, black line). By 1–2 h after infection, about 50% of the intracellular complexes contained detectable CA, and by 4 h after infection, less than 30% contained detectable CA. The kinetics of uncoating of the Q63A/Q67A mutant proceeded, as predicted, more slowly than that of the WT virus (Figure 4, red line). At 1 and 2 h afterwards, most of the particles were positive for CA (Figure 4, middle). By 4 h after infection, however, the number of particles that stained positive for CA in the Q63A/Q67A mutant was about the same as for WT virus. On the other hand, the cores associated with the T54A/N57A CA mutant were uncoated more quickly than the cores associated with WT CA at the early time points post infection (Figure 4, blue line). While a similar association of CA with intracellular viral complexes was detected at the 0-h time point (immediately after infection); by 1 h after infection, only about 20% of the complexes had detectable CA. This proportion stayed about the same at 2 and 4 h (Figure 4, blue line). These data demonstrate that a failure to uncoat the bulk of CA from the RTC cannot explain the inability of each of the CA mutants to infect nondividing cells. Rather, it appears that at least one of the mutants uncoats faster than WT virus.

In addition to the uncoating assay (Figure 4), we also used another assay that takes advantage of the fact that interaction of cyclophilin A (CypA) with viral CA enhances HIV-1 replication and that this enhancement is blocked by treatment with cyclosporine A (CsA). Since the target of CypA is the CA protein itself, we reasoned that the RTC would be sensitive to the negative effects of CsA for the amount of time that functional CA was associated with the RTC. Namely, the longer CA is associated with RTC, the longer CsA influences virus infectivity. We did this experiment in T cells that were infected with virus with its own envelope (X4 envelope) in order to more closely match the normal entry patterns of virus.

**Figure 5:**
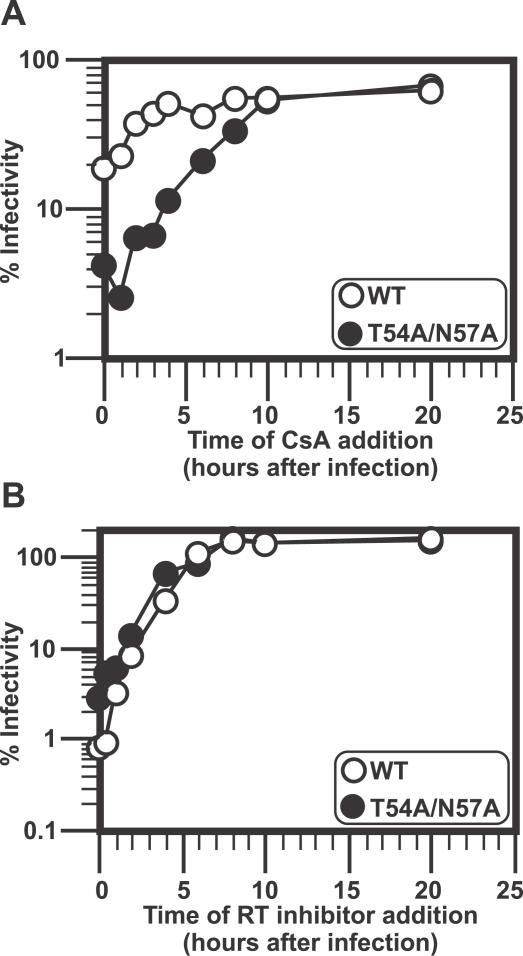
Kinetics of HIV-1 Sensitivity to CsA (A) CsA affects HIV-1 with the CA mutation T54A/N57A much longer than HIV-1 with WT CA. Human T cell Jurkat cells were infected with replication-competent HIV-1 virus encoding the WT envelope and the luciferase gene, which contains either the WT CA sequence or the T54A/N57A mutation. CsA was added to the well at indicated time points. Virus was added at 8 °C by spinolculation, and infections were synchronized by washing off the innocula and adding media at 37 °C. The relative percentage of infectivity was determined by assigning the luciferase value of the sample without CsA as 100% for each virus. White circles indicate WT, black circles the mutant (T54A/N57A). Similar results were observed in three independent experiments. (B) Reverse transcription (RT) of T54A/N57A progresses similarly to that of WT. Similar experiments were done as above (A), except that nevirapine (50 μM) was added instead of CsA.

A human T cell line, Jurkat cells, was infected with either WT HIV-1 or T54A/N57A, both of which are based on replication-competent viruses expressing luciferase. CsA was added at different times after inoculation of virus to see how long the effects of CsA on HIV-1 infection last. When the drug was added simultaneously with the initiation of infection of the viruses, WT HIV was moderately affected by the treatment of CsA (∼5-fold), whereas T54A/N57A was more severely restricted (∼25-fold) by the presence of the drug (Figure 5A).

The kinetics of the sensitivity of the two viruses to CsA appeared distinct. For WT virus, the sensitivity to CsA inhibition rapidly declined within the first few hours after infection (Figure 5A), and the half-maximal effect of CsA was seen at about 2 h. This result is in good agreement with the uncoating as for WT in Figure 4. The effects of CsA on the infection with T54A/N57A were more complex (Figure 5A). There was an increased inhibition of virus infectivity when CsA was added at 1 h after infection relative to when it was added at the same time as the infection (Figure 5A). This result was repeatable (*N* = 3). However, after that initial inhibition by CsA, there was a much slower gradual loss of CsA sensitivity, and the half-maximal effect was not observed until after 8 h.

As controls for the function of the RTC, we also measured the kinetics of resistance to a reverse-transcriptase inhibitor, nevaripine, and found that both WT and the T54A/N57A mutant had an equivalent kinetic sensitivity with a 50% maximal inhibition approximately 4 h after infection (Figure 5B). These results demonstrate that the T54A/N57A does not affect the extent (Figure 3), or kinetics (Figure 4), of reverse transcription. Nonetheless, the kinetics of CsA sensitivity of this mutant suggest that functional association of CA with infectious particles in T cells might occur with complex kinetics that could have a major effect on downstream events that are important for the ability to infect cells in a cell-cycle independent fashion.

## Discussion

Our previous study indicated that a chimeric HIV-1 having MLV CA fails to infect nondividing cells [21]. This suggests either that MLV CA negatively regulates virus infectivity in nondividing cells or that HIV CA is an essential factor for HIV infection of nondividing cells. Here, we provide direct evidence that HIV-1 CA is an essential factor since we identify mutations in CA that affect this process.

We previously proposed that inefficient disassembly of the viral core is responsible for the poor infectivity of MLV in nondividing cells [9] since there appears to be a difference in the amount of CA-associated MLV RTC and/or pre-integration complex (RTC/PIC) compared to that of HIV [14,19]. Thus, we expected mutants of HIV CA that failed to infect nondividing cells also to uncoat CA from the RTC less slowly than WT HIV. However, this does not seem to be the case (Figure 4). One of the mutants, Q63A/Q67A, behaved this way (Figure 4), but another mutant, T54A/N57A, appears to uncoat more rapidly than WT HIV (Figure 4). Thus, there appears to be no correlation between the uncoating of the bulk of CA soon after infection and the ability to infect cells independent of the cell cycle. It is also possible that CA mutations indirectly affect the rate of reverse transcription, which in turn could affect the composition of the PIC. However, we found no difference in either the amount of reverse transcription products made by the CA mutants studied here (Figure 3), or more importantly, no difference in the kinetics of completion of reverse transcription (Figure 5B).

However, all assays that look at total events soon after infection have the caveat that it is not possible to distinguish events leading to productive infection from events that are dead-end products. Thus, we also adapted an assay previously used to characterize CA association with RTC/PIC that relied on the kinetic sensitivity of HIV to the Trim-CypA restriction in owl monkey cells [34]. In this assay, only infectious events are scored, because the final read-out requires transcription from the provirus. We wished to use this assay in human T cells, and therefore relied on the kinetic sensitivity to cylophilin enhancement (i.e., decreased infectivity in the presence of CsA) rather than the restriction to Trim-CypA. We found that there is a functional association of CA with the RTC/PIC for several hours after infection (Figure 5) in Jurkat T cells. In addition, one of the CA mutants (T54A/N57A) was sensitive to CsA for a longer period of time than WT virus (on the order of 8–10 h), suggesting that T54A/N57A may retain some CA on the RTC for a longer period of time than WT (Figure 5). In contrast, the in situ uncoating assay found that this mutant uncoated faster than WT virus (Figure 4). Thus, an alternative interpretation is that the in situ assays detect the presence of an intact conical core structure, while the CsA sensitivity of HIV infection detects another function that is associated with a subset of CA that stays associated with the RTC for extended periods after uncoating. This could explain the increased sensitivity of the T54A/N57A mutant to CsA (Figure 5).

Another CA mutant (Q63A/Q67A) that lost the cell-cycle independence for infection was found to contain more CA proteins in the mature RTC in both the in situ uncoating assay (Figure 4), and in an independent biochemical assay [35]. These results support a model whereby some degree of association of CA with the RTC/PIC prevents infection of nondividing cells, and that total uncoating is the rate-limiting step for efficient infection of nondividing cells. Nonetheless, other models for a role of CA in other early steps of the virus life cycle that is important for cell-cycle independence cannot be ruled out.

The mechanistic basis of uncoating is not well understood, but the CA protein is the likely candidate for regulation of core disassembly due to differential levels of its association with intracellular virus complexes. Thus, it is possible that substitution of certain amino acid residues in the CA may alter the stability of virus cores and thus the kinetics of CA disassembly from the viral core in infected cells. However the cell-type specificity for the replication block of HIV CA mutants in nondividing cells argues against this possibility; if the intrinsic property of a virus by itself determines the reduced infectivity, then we would not likely see any difference in viral infectivity between different cell types. Instead, these data suggest the involvement of cellular factors in regulating the role of incoming viral cores in target cells.

Both MLV and HIV CA mutants require progression of cell cycle through mitosis for efficient replication (Figure 2). The long-held idea to explain the requirement of mitosis for productive infection by MLV is that MLV lacks the ability to transport its viral DNA into the nucleus across intact nuclear envelope of interphase cells and that mitosis literally removes this barrier of nuclear membrane because of nuclear membrane breakdown during mitosis [6]. Consistent with this idea, incorporation of tRNA has been implicated in the role of nuclear transport of HIV, and is absent in MLV [36]. However, we found that viral DNA of CA mutants is associated with the nucleus and results in the accumulation of 2-LTR circles. Thus, our data suggest that the HIV CA mutants only transduce cells that go through mitosis (Figure 2), but that nuclear import per se is not the limiting step. Indeed, others have also shown that HIV-1 CA can affect a post–nuclear entry step [35]. In addition, there is a report suggesting that factors other than the nuclear membrane govern the infectivity of MLV in nondividing cells (macrophages) [37]. Thus, while nuclear import of PICs is undoubtedly necessary during the viral life cycle, these data suggest that there are steps past nuclear entry that may be critical determinants for the ability to infect nondividing cells. The direct implication of CA in the infection of nondividing cells by HIV may help uncover novel means of disrupting this critical pathway in the viral life cycle.

## Materials and Methods

### Cell culture.

Cell lines were grown in DMEM supplemented with either 10% calf serum or fetal bovine serum (FBS). MDMs were prepared as described before [21] and cultured in RMPI with 10% FBS and 5% human serum.

### Viral constructs.

The proviral DNA pLai is an infectious molecular clone of HIV-1 [38]. Reporter virus constructs created based on pLai and encoding either the luciferase gene or the EGFP gene in place of the nef open reading frame were described previously (pLai3ΔEnv-luc2 and pLai ΔEnv-GFP3, respectively) [21]. They lack a functional envelope gene, allowing single-cycle replication assays by pseudotyping virions with envelope proteins supplied in trans. DNA fragment encompassing the HIV-1 CA-encoding region was digested from pLai with ApaI and BssHII and cloned into either pSL1180 or pBluescript II, both of which were also processed with the same combination of restriction enzymes. Point mutations were introduced in the plasmid DNA containing a part of HIV-1 sequence by using a commercial PCR mutagenesis kit. DNA constructs with a substitution were subject to DNA sequencing to ensure that any unwanted change is not made in the HIV-encoding fragment. DNA fragments encoding mutated CA sequences were cloned into ApaI/BssHII-digested, HIV-based reporter viruses (pLai3ΔEnv-luc2 and pLaiΔEnv-GFP3). HIV mutant reporter clones lacking the intact Vpr gene were created by combining pLaiΔEnv-GFP3 having the WT CA sequence or bearing a Q63A/Q67A mutation in the CA sequence with the previously reported Vpr mutant [39].

### Virus stocks.

Virus stocks were prepared by transient transfection of 293T cells with various combinations of DNA constructs. Transfections were performed with a commercial liposome-based transfection reagent (TransIT-LT1; Mirus). An expression vector of VSV G protein, pL-VSV-G, was used to make pseudotyped virions [40]. HIV-1 proviral DNA constructs encoding either luciferase or GFP were co-transfected with pL-VSV-G. Virus stocks for MLV were prepared by transfection of four DNA constructs: Gag-Pol vector, retroviral transfer vector, pL-VSV-G, and a construct for Tat expression (pCMV-tat: to allow efficient expression of VSV-G, which is under control of HIV-1 LTR promoter) [40]. Either pJK3 [40] or pCS2+mGP [21] was used as Gag-Pol expression plasmid construct. For GFP-encoding, MLV-based retroviral transfer vectors, either pLXCG or pLXSG were used [41]. For luciferase-encoding, MLV-based retroviral transfer vectors, pLNCluc was used [21]. Culture supernatant was harvested 2 and 3 d post transfection. The supernatant was cleared of cell debris by low-speed centrifugation and passed through 0.22-μm filters. Aliquots of the filtered supernatant were frozen at −80 °C. Supernatant of the virus stocks that exhibit a low titer in infectivity assay was concentrated by ultracentrifugation after low-speed spin and filtration. Concentrated viruses were also kept at −80 °C until use.

### Infectivity assays.

Infectivity of HIV CA mutants was compared between dividing and nondividing cells by single-cycle replication assays. Briefly, dividing HeLa or HOS cells were seeded at 8 × 10^5^ per well for 12-well plates or at 4 × 10^5^ per well for 24-well plates 1 d before infection. Nondividing cells were prepared by seeding three times more cells than the protocol for dividing cells and by treatment with 2 μg of aphidicolin per ml. The cells were infected with increasing amounts of virus stocks in the presence of 20 μg of DEAE-dextran per ml. Virus attachment was enhanced by spinoculation at 1,200*g* for 30 to 60 min at room temperature. Inoculated viruses were washed away and fresh medium were added to the plates. Two days after infection, infectivity was examined by enumerating GFP-positive cells with fluorescence-activated cell sorter for GFP-encoding viruses or by measuring the luciferase activity in lysed cells for luciferase-encoding viruses.

MDMs were infected with GFP-encoding virus stocks 9 to 10 d after preparation along with MT4, a human T cell line, in the presence of 20 μg of DEAE-dextran per ml. We used ten times more virus inocula for macrophage infection than for MT4 infection, as MDM is not as permissive as MT4 to HIV-1 infections. Infected cells were analyzed for GFP expression 2 to 3 d after infection.

### Cell-cycle analysis of virus-infected cells.

Experiments were based on a previous publication [23] but modified. Six 6-well plates containing one million HeLa cells per plate were prepared for each virus 1 d prior to infection. Virus inocula were chosen so that GFP-expressing cells will become 3% to 5% at 15 h after infection. Virus internalization and hence initiation of infection was synchronized by spinoculation at 1,200*g* for 30 min at 4 °C or 15 °C to prevent endocytosis of attached virions. Infection was initiated by placing the plates in humanized CO2 incubator at 37 °C. Cells were harvested at different time points and fixed with 1% paraformaldehyde in PBS overnight. The cells were then permeablized with 70% EtOH in PBS overnight and stained with 50 μg/ml of propidium iodide in the presence of 180 U of RNase A at 37 °C for 30 min. Analysis of DNA content and GFP expression was performed on a FACScalibur instrument (Becton Dickinson) by using CellQuest Pro software (Becton Dickinson). GFP fluorescence was determined by fluorescence emission at 530 nm in log amplification after excitation at 488 nm. PI fluorescence was measured with a filter that collects fluorescence above 590 nm. Doublets were excluded from the analysis by using the PI fluorescence area and the pulse width. Samples were analyzed until at least 5,000 GFP-positive cells were collected.

### Real-time PCR.

Subcellular fractionation was performed as described previously [8]. Genomic DNA was extracted from infected cells by using the QIAamp DNA Blood Mini kit (Qiagen). Copy numbers of viral DNA were measured by real-time PCR based on previous reports with minor modifications [31].

### Western blotting analysis.

Western blotting analysis of lysates and extracts obtained from subcellular fractionation was done as described previously [8]. Transferred membranes were probed with the following antibodies: mouse monoclonal anti-lamin B (101-B7; Calbiochem); sheep antibody against LDH I (Cortex Biochem).

### In situ uncoating assay.

Virus was generated by cotransfecting 7.4 μg HIV-1Δenv plasmids with WT CA or the CA mutants, 6.4 μg S15-mCherry to stain the viral membrane [32], 4.2 μg VSV-g, and 0.5 μg GFP-Vpr [33] to stain total viral particles into 10-cm plates of 293T virus-producing cells using polyethylenimine (PEI) (MW 25000, Polysciences). S15-mCherry is a fluorescent fusion protein that contains the 15 N-terminal amino acids of the cellular Src protein. This 15–amino acid sequence possesses a myristoylation sequence followed by a basic stretch of amino acids that is sufficient to cause membrane association and virion incorporation of the S15-mCherry protein [32]. As this membrane label is lost following fusion, this system can effectively discriminate between virions that have been non-productively endocytosed by the target cells (S15+, GfpVpr+) from those that have productively entered the host cell cytoplasm (S15−, GfpVpr+).

Virus was harvested 48 h post transfection and filtered through a 0.22-μm filter. For infections, HeLa target cells were seeded on fibronectin-treated coverslips and spinoculated for 2 h at 17 °C in the presence or absence of Bafilomycin A (Sigma). Bafilomycin A (BafA) was prepared in DMSO and diluted to a final concentration of 20 nm in DMEM. Virus was then removed and replaced with 37 °C media in the presence or absence of BafA, shifted to 37 °C, and fixed at the indicated time point post infection. HeLa cells on glass coverslips were fixed with 3.7% formaldehyde (Polysciences) in 0.1 M Pipes buffer (pH 6.8). Coverslips were stained with anti-p24 mAb AG3.0 (NIH AIDS Research and Reference Reagent Program) in blocking solution (10% normal donkey serum [Jackson ImmunoResearch Laboratories], 0.1% Triton X-100, 0.01% NaN_3_) for 30 min at RT for primary stain and secondarily stained with labeled Cy5 donkey anti-mouse antibodies (Jackson ImmunoResearch Laboratories). Coverslips were mounted on slides with Gel Mount (Biomedia). Images were collected and deconvolved with a Deltavision microscope and software (Applied Precision). Following deconvolution, the number of GFP-positive virions was assessed at each time point and each virion was individually inspected for punctate mCherry fluorescent signal and p24 Cy-5 signal.

### Time-course experiments with CsA.

Jurkat cells were seeded in 96-well plates at 100,000 cells per well. Virus inocula that produce approximately 100,000 relative light units in luciferase assays were used to inoculate the cells. Infections were enhanced by addition of 20 μg of DEAE-dextran per ml and by a brief spinoculation. Virus-containing medium was washed away and fresh medium was added at the start of infection. CsA resuspended in DMSO was added at various time points after infection. The infected cells were lysed in a cell culture lysis buffer (Promega) at 48 h after infection and luciferase activity of the lysates was measured using the Luciferase Assay System (Promega).

## Abbreviations

CA: capsid
CypA: cyclophilin A
CsA: cyclosporine A
GFP: green fluorescent protein
MDM: monocyte-derived macrophage
MLV: murine leukemia virus
PIC: pre-integration complex
RTC: reverse transcriptase complex
WT: wild-type
